# Integrating rehabilitation into health systems: A comparative study of nine middle-income countries using WHO’s Systematic Assessment of Rehabilitation Situation (STARS)

**DOI:** 10.1371/journal.pone.0297109

**Published:** 2024-02-05

**Authors:** Pauline Kleinitz, Carla Sabariego, Gwynnyth Llewellyn, Elsie Taloafiri, Ariane Mangar, Rabindra Baskota, Kedar Marahatta, Shiromi Maduwage, Myo Hla Khin, Vivian Wonanji, George Sampa, Ali Al-Rjoub, Jaber Al-Daod, Alarcos Cieza

**Affiliations:** 1 Sensory Functions, Disability and Rehabilitation Unit, Department for Noncommunicable Diseases, World Health Organization, Geneva, Switzerland; 2 Faculty of Health Sciences and Medicine, University of Lucerne, Lucerne, Switzerland; 3 Swiss Paraplegic Research, Nottwil, Switzerland; 4 Center for Rehabilitation in Global Health Systems, WHO Collaborating Center for Rehabilitation in Global Health Systems, University of Lucerne, Luzern, Switzerland; 5 School of Health Sciences, The University of Sydney, Sydney, Australia; 6 WHO Collaborating Centre for Strengthening Rehabilitation Capacity in Health Systems, Sydney, Australia; 7 Disability and Inequity Stream Leader, Centre for Disability Research and Policy, Sydney, Australia; 8 NHMRC Centre of Research Excellence Disability and Health, Melbourne, Australia; 9 Physiotherapy, Rehabilitation Division, Ministry of Health and Medical Services, Honiara, Solomon Islands; 10 Rehabilitation Department, Ministry of Health, Georgetown, Guyana; 11 Leprosy Control and Disability Management Division, Ministry of Health and Population, Kathmandu, Nepal; 12 Non-communicable Disease Department, World Health Organization Country Office, Kathmandu, Nepal; 13 Youth, Elderly, Disability Unit, Ministry of Health, Nutrition and Indigenous Medicine, Colombo, Sri Lanka; 14 Department of Physical Medicine and Rehabilitation, Yangon General Hospital, University of Medicine, Yangon, Myanmar; 15 Curative Services Division, Ministry of Health, Community Development, Gender, the Elderly and Children, Dodoma, Tanzania; 16 Disease Prevention and Control Division, Ministry of Health, Lusaka, Zambia; 17 Physical Medicine and Rehabilitation Focal Point, Ministry of Health, Amman, Jordan; State Health Resource Center, Chhattisgarh, INDIA

## Abstract

**Background and objective:**

The need for rehabilitation is growing due to health and demographic trends, especially the rise of non-communicable diseases and the rapid ageing of the global population. However, the extent to which rehabilitation is integrated into health systems is mostly unclear. Our objective is to describe and compare the nature and extent of integration of rehabilitation within health systems across nine middle-income countries using available Systematic Assessment of Rehabilitation Situation (STARS) reports.

**Methods:**

Cross-country comparative study with variable-oriented design using available rehabilitation health system assessment reports from nine middle income countries.

**Findings:**

The integration of rehabilitation into health systems is limited across countries. Governance and financing for rehabilitation are mostly established within health ministries but weakly so, while health information systems are characterized by no available data or data that is insufficient or not routinely generated. The overall numbers of rehabilitation workforce per capita are low, with frequent reports of workforce challenges. In most countries the availability of longer-stay, high-intensity rehabilitation is extremely low, the availability of rehabilitation in tertiary hospitals is modest and in government supported primary care its almost non-existent. Multiple concerns about rehabilitation quality arose but the lack of empirical data hinders formal appraisal.

**Conclusion:**

The study sheds light on the limited integration of rehabilitation in health systems and common areas of difficulty and challenge across nine middle income countries. All countries were found to have a basis on which to strengthen rehabilitation and there were often multiple areas within each health system building block that required action in order to improve the situation. Findings can inform governments, regional and global agencies to support future efforts to strengthen rehabilitation. Additionally, our study demonstrates the value of STARS reports for health policy and systems research and can serve as a model for further comparative studies.

## Introduction

Rehabilitation is a key health strategy that optimizes population functioning [1], and contributes to better health and wellbeing of populations [2]. The need for rehabilitation is growing, associated with health and demographic trends, especially the increase of non-communicable diseases (NCD) and rapidly ageing populations. Data from the 2019 Global Burden of Disease (GBD) study indicates 2.4 billion people are living with health conditions that could benefit from rehabilitation [3]. People with unmet rehabilitation needs frequently experience greater difficulties moving, communicating, caring for themselves and being productive, including people with disabilities who experience discrimination, social exclusion and humans rights abuses [4]. Nevertheless, in many low and middle income countries (LMICs) rehabilitation provision is very limited and much of the population needs go unmet [5]. In many LMICs, rehabilitation is often an under-developed and under-valued area of health care [6], and the COVID-19 pandemic also unveiled its weakness as rehabilitation was one of most highly disrupted services [7]. To address the many challenges faced by rehabilitation, especially in LMICs, WHO launched the Rehabilitation2030 initiative in 2017 [8] and is developing guidance documents and tools to support countries strengthening rehabilitation in their health systems.

From its outset, the Rehabilitation2030 initiative emphasised the need to integrate rehabilitation at all levels of health care within health systems to ensure a continuum of care that is in line with people’s needs across the life course [9]. Building sustainable rehabilitation services at all levels of care necessitates its integration across the many components of a health system, for example its planning, financing, workforce and information systems [10]. Evidence on the nature and the extent to which rehabilitation is included in health systems is scarce. This knowledge, however, is important for understanding how to organise national and international efforts to strengthen rehabilitation. For WHO and other development partners this can inform the development of programmes and projects, as well as guidance and resources for country support.

Acknowledging the need for evidence and that strengthening efforts for rehabilitation need to be tailored to national needs and priorities, WHO developed a health system assessment (HSA) tool [11], the Systematic Assessment of Rehabilitation Situation (STARS). STARS generates standardised and structured assessments [12] and is part of the WHO Rehabilitation Guide for Action [11], an online available resource that guides rehabilitation strategic planning. STARS allows for cross-country comparative studies, a type of health policy and systems research (HPSR) [13], about the nature and extent to which rehabilitation is integrated in health systems at national, regional and global levels. Such studies provide information about the current situation and areas for improvement [14]. Additionally, governments can learn from countries with similar experiences in developing rehabilitation in health systems.

The objective of this paper is therefore to describe and compare rehabilitation status across nine middle-income countries using available STARS reports, exploring the policy and programme implications of the findings. Our paper contributes to filling the lack of evidence relevant to governments, regional and global agencies to support future efforts to strengthen rehabilitation in health systems and serves as a model for future HPSR research on rehabilitation.

## Methods

Cross-country comparisons can be categorised into case-oriented or variable-oriented studies [15]. We used a broad variable-oriented design, which is a form of HPSR [13] that supports generation of hypotheses and can be used to identify the differences across countries.

### STARS

STARS was developed between 2016–2019 by WHO. The development of STARS, and its assessment components, has been described elsewhere [16], and the product itself, including the definitions and descriptions of all the assessment components and how to use STARS is part of the WHO Guide for Action and available online [11]. STARS is an HSA tailored to rehabilitation. It is made up of three components; a Manual which guides the assessment process and preparation of reports; a Template for Rehabilitation Information Collection (TRIC) which guides the initial collection of data and information from countries; and a Rehabilitation Maturity Model (RMM) which defines 50 components for assessment and describes these on a 4-level maturity continuum. STARS utilises a logic model to its structure whereby the health system comprises building blocks (HSBBs) which cover areas of inputs (governance, financing, information, medicines and health products, workforce) and outputs (services). Assessment of services considers what is available at all levels of tertiary to primary healthcare (PHC) and in the community, as well as its quality, such as effectiveness and person-centred characteristics. The logic model prompts descriptions of service outcomes (at individual and population levels) and the extent of attributes of a well-functioning health system such as equity, efficiency and sustainability that are present. STARS generates a tailored report that is primarily designed to inform national stakeholders and make recommendations on how to strengthen rehabilitation in national health systems.

### Countries

The 10 countries from the six WHO regions that were ‘early adopters’ implementing WHO STARS between August 2019 and February 2021 were eligible to participate. Their WHO STARS implementations resulted in final reports endorsed by government and publicly available in English. A request to use the information within STARS reports for this study was sent to the rehabilitation focal points of the ministries of health (MOH), all of whom had engaged in drafting of the original STARS report. After obtaining their written permission, the data was extracted from reports and included in the study. Approval for the field testing of STARS in these countries was received from the WHO Ethics Committee.

### Data extraction

The first author (PK) initially mapped the common assessment components within STARS reports to the six HSBBs. All items that had either a quantifiable (e.g., workforce numbers) or empirical/observable feature (e.g., rehabilitation plan), and were similarly defined, measured or described across STARS reports were selected. Outcomes and attributes components were initially considered but not included because they lacked the quantifiable and empirical data: an example are measures for effective coverage which were not available in all countries.

The extracted data for each country was then sent to the rehabilitation focal points of the MOH and corresponding country co-author(s), who reviewed it for accuracy and precision, edited any inaccurate or imprecise information and added further or missing details. This step served as validation of the extracted information, noting that an earlier validation had also occurred during drafting and country review of the original STARS report. Where countries lacked the required information in their STARS report it was noted as ‘not available’.

### Data analyses

Country data was compiled into a common Microsoft Excel file. For qualitative items, data is presented in tables. For quantitative items, numbers or percentages (as required) were included and descriptive statistics (mean, median and range) were derived whenever possible. Where needed, quantitative data were standardised relative to population size. Analyses were conducted to 1). Identify differences across countries; and 2). Identify similarities and differences between countries accordingly to income categorisation grouping (either lower middle income or upper middle-income). The policy and programme implications of the findings are included in the discussion.

## Results

Nine middle income countries (Table 1) agreed to participate, one from each WHO region: three upper middle-income countries namely Georgia, Guyana and Jordan; and, six lower middle-income countries namely Nepal, Myanmar, Solomon Islands, Sri Lanka, Tanzania and Zambia. No low-income countries were ‘early adopters’ of the WHO STARS and therefore not included in the study.

**Table 1:** Characteristics of participating countries. Current Health expenditure per capita and GDP per capita in current international $. Income status: lower or upper middle income.

Country	Year STARS finalized	WHO Region	Population	Life expectancy	Current Health expenditure per capita	GDP per capita	World Bank Income group
**1. MYANMAR**	2019	SEARO	53,709,000	66.8	291.74	5,121.90	Lower Middle Income
**2. SRI LANKA**	2019	SEARO	21,229,000	75.3	516.92	13,219.70	Lower Middle Income
**3. NEPAL**	2020	SEARO	28,096,000	70.2	180.41	4,007.00	Lower Middle Income
**4. SOLOMON ISLANDS**	2019	WPRO	653,000	71.1	107.63	2,618.10	Lower Middle Income
**5. UNITED REPUBLIC OF TANZANIA**	2020	AFRO	56,313,000	63.9	112.46	2,780.10	Lower Middle Income
**6. ZAMBIA**	2019	AFRO	17,352,000	62.3	208.44	3,456.30	Lower Middle Income
**7. GEORGIA**	2020	EURO	4,003,000	72.6	795.9	14,761.50	Upper Middle Income
**8. JORDAN**	2018	EMRO	9,965,000	74.3	737.75	10, 351.2	Upper Middle Income
**9. GUYANA**	2019	PAHO	779,000	66.2	512.92	19,697.20	Upper Middle Income

Altogether 49 STARs items were selected for the comparison. A small number of items, such as expenditure, had limited available or comparable information across countries but were included due to their relevance for scaling up rehabilitation.

### Rehabilitation governance (Tables 2 and 3)

In all nine countries, MOH was primarily responsible for rehabilitation, however in Georgia the social affairs section of the joint health and welfare ministry led rehabilitation and in Jordan multiple agencies contributed and MOH was not the primary agency (Table 2). Only Nepal, Tanzania and Zambia had a regular leadership and coordination mechanism convened by MOH. Georgia, Guyana, Nepal, Solomon Islands, Sri Lanka, Tanzania and Zambia had rehabilitation focal points within the MOH with responsibility for rehabilitation; Jordan and Myanmar MOHs relied on rehabilitation professionals located within health services for governance of rehabilitation. Six countries had addressed rehabilitation in their National Health Strategic Plan and all countries had included rehabilitation in at least one other health plan, most frequently in the health plan for NCDs. All countries reported that rehabilitation was included in their existing health policy and/or legislation although not always explicitly mentioned; additionally, 5 countries explicitly addressed rehabilitation in their disability legislation (Table 3). Annual routine reporting on availability of rehabilitation services in government healthcare occurred in two countries. Leadership, coordination and planning for assistive technology (AT) was generally characterised as limited and fragmented. Nepal had a government convened multi-stakeholder AT working group, and Nepal and Sri Lanka had developed national Assistive Product Priority Lists (APPL). No observable distinction in leadership and governance occurred between the upper and lower middle-income countries.

**Table 2:** Rehabilitation governance.

COUNTRY	Government agency charged with rehabilitation leadership	Rehabilitation capacity within government administrative structure, both human and financial	Mechanism for national rehabilitation coordination (committee, platforms, working groups etc)	Rehabilitation addressed within health policy or legislation	Rehabilitation within National Health Strategic Plan (NHSP)
**1. MYANMAR**	Ministry of Health and Sports (MOHS)	No single designated focal unit within MOHS administrative structure; both Departments of Medical Services and Public Health play a role; No dedicated officer, ad hoc support provided through the Public Health’s NCD unit. No recurrent annual budget.	Myanmar Society for Rehabilitation Medicine plays strong coordination role and meet quarterly. The Society works closely with MOHS but is not an official government mechanism and its mandate is not cross-sectorial	Addressed within health policy although not explicitly; included in the Disability legislation (Law passed in 2015, Bylaws passed in 2017).	No. However, the Myanmar National Health Plan 2017–2021 had broad framework featuring few specific areas of healthcare, it was focused on the health system building blocks
**2.** **SRI LANKA**	Ministry of Health, Nutrition and Indigenous Medicine (MOHNIM)	Designated focal unit within MOHNIM—Youth, Elderly, Disability Unit with dedicated officer for rehabilitation. Recurrent annual budget allocated.	Committee exists under mandate of National Disability Plan but were not operating. Coordination meetings occur ad hoc for specific projects.	Included but not explicitly addressed.	Yes. Included in the National Strategic Framework for Development of Health Services 2016–2025
**3.** **NEPAL**	Ministry of Health and Planning (MOHP)	Designated focal unit within MOHP—Leprosy Control Disability Management Section, with dedicated officer for rehabilitation. Recurrent annual budget allocated.	A working group sits under the leadership of the MOHP, created after the development of the MOHP led Disability Management (Prevention, Treatment and Rehabilitation) Strategy and Action Plan (DMPTRSAP). MOHP convenes meetings quarterly.	Included in both health policy and legislation. Also included in disability legislation	Yes. Included in the National Strategic Framework for Development of Health Services 2016–2021 with its own objective.
**4. SOLOMON ISLANDS**	Ministry of Health and Medical Services (MHMS)	Designated focal unit within MHMS—Rehabilitation and Physiotherapy Division, with dedicated officer for rehabilitation. Recurrent annual budget allocated.	No official mechanism, focal unit in MHMS undertaking the coordination role as needed.	Included within the MHMS Role Delineation Policy.	Yes, briefly. Referred to in the NHSP 2016–2020 but no objective; however, this plan addresses broad themes such as coverage, quality and partnerships.
**5. TANZANIA**	Ministry of Health, Community Development, Gender, the Elderly and Children (MOHCDGEC)	Focal officer for rehabilitation within the NCD Section under directorate of Curative Services Division of the MOHCDGEC. Recurrent annual budget allocated.	The MOHCDGEC focal point for rehabilitation chairs a quarterly Rehabilitation Technical Working Group meeting. Membership includes multiple stakeholders and the Ministry of Social Welfare & President’s Office	Included in the National Health Policy (2017).	Yes, briefly. No objective, included within other areas such as NCDs, mental health and disability.
**6.** **ZAMBIA**	Ministry of Health (MOH)	Rehabilitation designated within the Directorate of Clinical Care and Diagnostic Services, has a coordinator and a technical working group. No recurrent annual budget for rehabilitation.	Recent creation of a Rehabilitation Technical Working Group, convened by MOH; meets quarterly.	Included, explicitly mentioned.	Yes, included.
**7.** **GEORGIA**	Ministry of Internally Displaced Persons from the Occupied Territories, Labour, Health and Social Affairs (MoIDPLHSA)	MoIDPLHSA policy department has a focal officer for rehabilitation, as does the Social Protection Policy Unit. Leadership from social programmes for people with disabilities. Recurrent annual budget allocated.	No official mechanism. Project based working groups in place.	Not explicitly, although some health policy and legislation applicable. Rehabilitation addressed in the disability law (not yet approved).	Not included in the current health plan—State Concept of Healthcare System of Georgia for Universal Health Care and Quality Control for the Protection of Patients’ Rights 2014–2020
**8.** **JORDAN**	No central leadership. Ministry of Health (MOH) in charge of rehabilitation, Royal Medical Services (RMS) administration for RMS and University administration for university hospitals.	Designated focal unit in MOH—Directorate of Disabilities and Mental Health, however no dedicated rehabilitation officer exists. No recurrent annual budget for rehabilitation	No official mechanism, development partner supports coordination meetings, increased during development of strategic plan	Not explicitly addressed in health legislation. Included in disability legislation.	Not included within the NHSP.
**9.** **GUYANA**	Ministry of Public Health (MOPH)	Designated unit in MOPH—Department of Disability and Rehabilitation Services, with dedicated rehabilitation officer. Recurrent annual budget allocated.	No official mechanism nor routine meetings. Meetings that contribute to coordination occur ad hoc for specific projects.	Not explicitly addressed in health policy and legislation. Rehabilitation addressed in the disability law from 2009.	Yes. Included in the Vision2020 for health

**Table 3:** Rehabilitation governance, cont’d.

	Rehabilitation within other health plans	Rehabilitation strategic plan	Routine (e.g. annual) rehabilitation status reporting	Leadership, coordination and planning for assistive technology (AT)	Regulation for rehabilitation services and assistive technology *(See Table 4 for info about workforce regulation)*
**1. MYANMAR**	Yes. Included in MOHS NCD strategic plan and national Trauma Roadmap	No. (Have since developed)	No, and not part of National Public Health Statistics Report. Some information on status of rehabilitation (workforce, services) was available on request.	Limited previous MOHS leadership, coordination and planning, recently increasing. AT included in the National Disability Law. No Priority Assistive Product List.	No specific provisions for rehabilitation service regulation. No formal quality standards exist for AT nor regulation mechanisms.
**2.** **SRI LANKA**	Yes. Included in Elderly Healthcare Delivery Plan, briefly included in the NCD policy.	Rehabilitation included in National Disability Plan. Planning also occurred during development of the ’National guideline for rehabilitation services’.	Yes, some information included in the Annual Health Statistics Report, recently commenced monitoring and reporting on prosthetic and orthotic services.	Recent increase in MOHNIM leadership, coordination and planning, together with Ministry of Social Services and Primary Industries (MOSSPI). AT included in the National Disability Action Plan. Recent identification of an Essential Assistive Product List, now trying to underpin with government funding commitment.	No specific provisions for rehabilitation service regulation. No formal quality standards exist for AT nor regulation mechanisms.
**3.** **NEPAL**	Yes. Included within 2 sets of National Minimum Standards for tertiary and secondary hospitals, and for basic services.	Rehabilitation included in the MOHP led Disability Management (Prevention, Treatment and Rehabilitation) Strategy and Action Plan (DMPTRSAP).	Not occurring.	Some previous MOHP leadership, coordination and planning, recently MOHP convening an assistive product technical working group for AT coordination and planning. AT included in the MOHP DMPTRSP. Recent identification of a Priority Assistive Product List.	No specific provisions for rehabilitation service regulation. No formal quality standards exist for AT nor regulation mechanisms.
**4. SOLOMON ISLANDS**	Yes, included in MHMS Role Delineation Policy and NCD Strategic Plan.	No. (Have since developed)	Limited routine reporting (mostly personnel numbers) through regular MHMS reports.	MOHMS leadership, coordination and planning has occurred for many years, the focal unit is the Rehabilitation and Physiotherapy Division—historically focused on mobility and environment products. No Priority Assistive Product List has been developed.	No specific provisions for rehabilitation service regulation. No formal quality standards exist for AT nor regulation mechanisms.
**5. TANZANIA**	Yes, included in both the Strategic Plan for NCDs and Eye Health. Also included in National Basic Standards for Health Facilities, Volume 6.	No (Have since developed)	Not occurring	Limited previous MOHCDGEC leadership, coordination and planning. No Priority Assistive Product List has been developed.	No specific provisions for rehabilitation service regulation. No formal quality standards exist for AT nor regulation mechanisms.
**6.** **ZAMBIA**	Yes, included in mental health, eye health and other programme areas.	No.	Not occurring	Some MOH leadership, coordination and planning, the focal person is the rehabilitation officer within the Directorate of Clinical Care and Diagnostic Services, historically focused on mobility products. No Priority Assistive Product List has been developed.	No specific provisions for rehabilitation service regulation. No formal quality standards exist for AT nor regulation mechanisms.
**7. GEORGIA**	Rehabilitation included in Early Childhood Intervention decree, plan and programmes.	No.	Not occurring	Some leadership, coordination and planning, especially linked to the guidance for provider pricing and supplier criteria for the MoIDPLHSA programme. Leadership for AP has sat within the Social Protection section of MoIDPLHSA. No Priority Assistive Product List has been developed.	There is a system of approved service providers for MoIDPLHSA funded AT and children’s rehabilitation services. MoIDPLHSA provides some technical specifications for AT. No formal AT quality standards nor regulation mechanisms exist.
**8.** **JORDAN**	Yes. Included in Mental Health and Substance Abuse Plan	No. (Have since developed)	Not occurring.	Limited leadership, coordination and planning between MOH, Ministry of Social Development and Ministry of Education, all of which provide AT. No mechanisms in place for centralised planning or coordination. No Priority Assistive Product List has been developed.	A license is required from the MOH to have a private practice. No other specific provisions for rehabilitation service regulation. No formal quality standards exist for AT nor regulation mechanisms.
**9. GUYANA**	Yes. Included in the NCD and PHC Plan, also within the Package of Essential Health Services.	Yes, included in a Disability and Rehabilitation Strategic Plan 2014–2020, developed by MOPH. (New plan now being developed)	Occurring internally in MOPH on an annual basis, reports the number of new patients and major initiatives.	Leadership is from MOPH, some planning and coordination, the focal unit is the Department of Disability and Rehabilitation Services—historically focused on mobility and environment products. No Priority Assistive Product List has been developed.	No specific provisions for rehabilitation service regulation. No formal quality standards exist for AT nor regulation mechanisms.

### Rehabilitation financing (Tables 4 and 5)

All countries financed rehabilitation using multiple sources, typically a combination of government tax-based public health or social health insurance schemes (as well as other insurance schemes in some instances), individuals’ out of pocket (private) contributions and development partners. Eight countries included rehabilitation in their major tax-based public health financing scheme in which the MOH is main funder and provider; Georgia which was the exception included rehabilitation in their financing scheme for services for people with disabilities (Table 4). Five countries described having a basic or essential package of health services (typically financing for services in primary care and lower end of secondary care), that is, Georgia, Guyana, Myanmar, Nepal and Sri Lanka; of these only Guyana and Sri Lanka included rehabilitation in this package of health services. Rehabilitation expenditure data was mostly not available or incomplete, hence expenditure on rehabilitation as proportion of total health expenditure was not available for comparative analysis (Table 5). Where expenditure on rehabilitation data existed, it was lowest at 0.2% (Nepal) and highest at 1.2% (Guyana). Out of pocket expenditure (OOPE) appeared to be a significant contribution to financing rehabilitation but detailed data was not available. The combination of government tax-based public health or social health insurance schemes financing rehabilitation suggested that the level of population coverage was high however access to services was low because of scarcity and concentration of rehabilitation in major urban centres. Across all countries, Assistive Products (AP) financing was limited and OOPE for AP was considered substantial, typically more than for other rehabilitation services. Fees-for-service (a form of OOPE that the provider is reimbursed for with each service provided) existed for government funded rehabilitation in most countries: in Guyana, Myanmar, Solomon Islands, Sri Lanka and Zambia these fees were characterised as minimal.

**Table 4:** Rehabilitation financing.

COUNTRY	Availability of rehabilitation expenditure data	Estimated percentage of rehabilitation as total health expenditure (THE)	Key sources of financing for rehabilitation	Rehabilitation included in the Essential/Basic Healthcare Packages (where they exist)
**1. MYANMAR**	Not available routinely but can be made available through additional analysis commissioned at country level.	0.6% of total health expenditure, inclusive of government and development partner rehabilitation expenditure.	Included in major tax-based public health scheme in which MOHS is main funder and provider; also included in statuary health insurance scheme for government and private international employees, which covers approx. 1% of population; Private health sector rapidly expanding with rehabilitation provision; 51% of Total Health Expenditure (THE) is out-of-pocket costs (OOP), no rehabilitation specific data available; development partners fund small number of local NGOs delivering rehabilitation in community settings and also contribute to assistive products, including those delivered in government hospitals. Unknown details of contribution, characterised as low.	Rehabilitation is not included in the MOHS Essential Package of Health Services. Inclusion planned in the future.
**2.** **SRI LANKA**	Not available.	Not available.	Included in major tax-based direct scheme in which MOHNIM is main funder and provider. The Ministry of Social Services and Primary Industries (MOSSPI) contributes to assistive product financing for people with disabilities through a disability allowance programme that includes healthcare costs. Private health sector rapidly expanding, rehabilitation services included. 38% of Total Health Expenditure is OOP costs, no rehabilitation specific data. Development partners fund small number of local NGOs delivering rehabilitation mostly in community settings. They also fund assistive products, including those delivered in government hospitals. Unknown details of contribution, characterised as low.	Rehabilitation is included in the MOHNIM Sri Lanka Health Services Package (SLESP)
**3. NEPAL**	Some data available through National Health Accounts (NHA), which includes MOHP and external development partner contribution, but not the funds of MWCSC, nor rehabilitation service costs that are embedded in health facility budget lines, such as rehabilitation personnel and equipment.	0.2% of THE, based on Nepal’s National Health Accounts.	Included in major tax-based public health scheme in which MOHP is main funder and provider. Included in National Health Insurance Scheme, which approx. 10% population is enrolled. Included in a ’Special Fund for significant health conditions’, currently eight conditions. Included in small number of conditional grants by MOHP and MWCSC to private institutions that deliver rehabilitation, e.g. National Spinal Injury Rehabilitation centre and physical rehabilitation centres. Large private health sector and continues to expand, rehabilitation included. 60% of THE are OOP costs, no rehabilitation specific data available, however because significantly large portion of rehabilitation located in private sector likely very high OOP. Development partners fund some local NGOs delivering rehabilitation in not-for-profit hospitals and community settings. They also contribute to rehabilitation in the MOHP Sector Wide Approach, and within this the foreign sources for rehabilitation made up 95% of the total rehabilitation expenditure.	Rehabilitation is not included in the MOHP Basic Healthcare Package.
**4. SOLOMON ISLANDS**	Some data available through MHMS 2018 Budget. Includes the budget allocated for activities conducted by MHMS administrative structure and assistive products procurement. Does not include the rehabilitation worker salaries in government hospitals and services.	0.3% of THE, based on 2018 MHMS budget.	Included in major tax-based direct scheme in which MHMS is main funder and provider. Private health sector is very small. 3–4% of THE are OOP costs, no rehabilitation specific data available. Development partners fund small number of local NGOs delivering rehabilitation in community settings, they also contribute to a significant proportion of the assistive products provided both in government healthcare and through NGOs.	No Essential/Basic Package has been defined.
**5. TANZANIA**	Not available.	Not available.	Included in major tax-based public health scheme in which MOHCDGEC is main funder and provider. Also included in the National Health Insurance Fund (NHIF), which covers approx. 8% of the population. There is also the Improved Community Health Fund (ICHF) which covers the informal sector and rehabilitation can be included. Private health sector is steadily expanding, rehabilitation included. 24% of THE are OOP costs, no rehabilitation specific data available. Development partners fund a number of local NGOs delivering rehabilitation in both not-for profit hospital and community settings, they also contribute towards assistive products. Unknown details of contribution, characterised as low.	No Essential/Basic Package has been defined.
**6.** **ZAMBIA**	Not available. A separate MOH budget allocated for procuring assistive products was available.	Not available.	Included in major tax-based public health scheme in which MOH is main funder and provider. Approx. 4% of population have private health insurance and rehabilitation included in these schemes. Private health sector remains modest but expanding, rehabilitation included. 10% of THE are OOP costs, no rehabilitation specific data available. Development partners fund some local NGOs delivering rehabilitation in community settings, they also contribute to assistive products. Unknown details of contribution, characterised as low.	No Essential/Basic Package has been defined.
**7. GEORGIA**	Some data available. MoIDPLH Social rehabilitation and childcare programme allocates annual rehabilitation budget under early childhood development, assistive products, and rehabilitation.	The rehabilitation budget is estimated to be 0.31% of the MoIDPLH budget, this amount is for the rehabilitation scheme for eligible (disabled) population	Included in the (tax-based) MoIDPLH Social rehabilitation and child care programme scheme which funds services for eligible population groups using service packages and vouchers. Local government budgets for disability can allocate funds towards rehabilitation. Notably, not included in the Universal Health Coverage Programme (UHCP) which is the major source of health financing. A range of voluntary health insurance (VHI) schemes exist, these commonly include rehabilitation. Highly privatised health service structure. 55% of THE are OOP costs, no rehabilitation specific data however because rehabilitation is not included in the UHCP its likely very high OOP rehabilitation costs. Development partners fund range of initiatives, including for early childhood intervention, rehabilitation centres and prosthetic and orthotics training, infrastructure and services. Unknown details of contribution, characterised as low to moderate.	The Universal Health Coverage Programme (UHCP) defines services for primary care and does not include rehabilitation.
**8.** **JORDAN**	Not available.	Not available.	Included in the major tax-based public health scheme in which services are provided through MOH, Royal Medical Services and University Hospital system. Also included in the health insurance schemes, of which approx. 68% of the population is included. Rehabilitation also included in the services provided for people with disabilities through the Ministry of Social development and the Ministry of Education. Private health sector is steadily expanding with rehabilitation included. 30% of THE are OOP costs, no rehabilitation specific data. Development partners fund small number of local NGOs delivering rehabilitation in community settings, they also contribute to assistive products. Unknown details of contribution, characterised as low.	No Essential/Basic Package has been defined.
**9. GUYANA**	Yes. Available through the MOPH annual budget, it reflects expenditure within MOPH administrative structure and all the rehabilitation worker salaries, but not equipment and consumables that are part of hospital/facility budgets.	1.2% of THE, inclusive of all the government rehabilitation workers and equipment costs, based on annual MOPH budget.	Included in tax-based public health scheme in which MOPH is main funder and provider. Also included in National Insurance Scheme (NIS) that covers workers in public and private sector. The Ministry of Social Protection and Indigenous Affairs contributes to assistive product financing. Private health plays significant role with rehabilitation included. 37% of THE are OOP costs, no rehabilitation specific data available. Development partners fund local NGOs delivering rehabilitation in community settings. They also contribute to assistive products, including those delivered in government hospitals. Other international contributions have supported training and equipment. Unknown details of contribution, characterised as low overall but significant for AP.	The Package of Essential Health Services exists and rehabilitation is included, however AP is not.

**Table 5:** Rehabilitation financing, cont’d.

	Fee, payment and additional information about rehabilitation costs in government healthcare	Levels of population coverage for rehabilitation—by major health/social financing schemes	Types of rehabilitation services covered within the rehabilitation financing mechanism
**1.** **MYANMAR**	No or minimal fees exist for rehabilitation in government healthcare. Some fees exist for AP.	The tax-based public health scheme of MOHS covers Myanmar citizens, in this way coverage is good. Insurance schemes have low level of coverage, currently at 1%	The tax-based public health scheme finances the rehabilitation provided in government hospitals and services, this includes the full scope of interventions delivered by existing rehabilitation workers. Service caps do not exist however the large rehabilitation workload (commonly 1–2 therapist per 100 beds in tertiary hospitals), early discharge practices (because of hospital bed pressure), and limited uptake of outpatient appointments (client transport costs deter), significantly limits the dosage of rehabilitation provided. Assistive products also included in tax-based direct scheme but limited range (mostly mobility) and quantity is provided. The statutory insurance scheme funds a similar scope of rehabilitation interventions in private hospitals and clinics however caps on number of sessions exist.
**2.** **SRI LANKA**	No or minimal fees exist for rehabilitation in government healthcare. Some fees exist for AP.	The tax-based public health scheme of MOHNIM covers Sri Lankan citizens, in this way coverage is good. MOSSPI funds are for people with disabilities who qualify under their scheme. Other private insurance schemes have low level of coverage.	The tax-based public health scheme finances the rehabilitation provided in government hospitals and services, this includes the full scope of interventions delivered by existing rehabilitation workers. Service caps do not exist however the large rehabilitation workload (commonly 1–2 therapist per 100 beds in tertiary hospitals), early discharge practices (due hospital bed pressure), and limited uptake of outpatient appointments (client transport costs deter), significantly limits the dosage of rehabilitation provided. Assistive products also included in tax-based direct scheme but limited in range (mostly for mobility products) and quantity supplied. MOSSPI funding through local government typically more flexible covers a wider range of assistive products.
**3.** **NEPAL**	A fee structure exists across all government healthcare. This includes means testing. Poorer population groups or people registered as a person with a disability can be exempt. The fees are itemised for each service received within hospital care. Some fees exist for AP.	The tax-based public health scheme of MOHP covers Nepali citizens, in this way coverage is good. The NHI scheme is relatively recent, its current 10% population coverage is expected to increase over time.	The tax-based public health scheme finances the rehabilitation provided in government hospitals and services, this includes the full scope of interventions provided by existing rehabilitation workers, however the total number and professions included in government healthcare is low. The NHI funds physiotherapy (only) and a small range of assistive products.
**4.** **SOLOMON ISLANDS**	No or minimal fees exist for rehabilitation in government healthcare. Some fees exist for AP.	The tax-based public health scheme of MHMS covers Solomon Island citizens, in this way coverage is good.	The tax-based public health scheme finances the rehabilitation provided in government hospitals and services, this includes the full scope of interventions provided by existing rehabilitation workers. Assistive products also included but limited in range (mostly mobility products) and quantity supplied, there are fees applied to AP but often not for other rehabilitation services.
**5. TANZANIA**	Fees exist for rehabilitation in government healthcare, the amount varies between facilities and services. Fee exemptions exist for children under 5yo, people over 60yo with low economic status and people with long-term disability.	The tax-based public health scheme of MOHCDGEC covers Tanzania’s citizens, in this way coverage is good. The NHIF covers 8% of population.	The tax-based public health scheme finances the rehabilitation provided in government hospitals and services, this includes the full scope of interventions provided by existing rehabilitation workers. Assistive products also included but limited in range (mostly mobility products) and quantity supplied, there are fees applied to AP. The NHIF covers rehabilitation consultation fees from a range of providers (e.g. physiotherapy and speech and language therapy) and a small range of assistive products.
**6.** **ZAMBIA**	No or minimal fees exist for rehabilitation in government healthcare. Some fees exist for AP.	The tax-based public health scheme of MOH covers Zambian citizens, in this way coverage is good.	The tax-based public health scheme finances the rehabilitation provided in government hospitals and services, this includes the full scope of interventions provided by existing rehabilitation workers. Assistive products also included but limited in range (mostly mobility products) and quantity supplied, and fees are applied to AP. Rehabilitation included in the private health insurance schemes.
**7.** **GEORGIA**	Fees exist for rehabilitation because it is not part of UHCP, only eligible people with disability under the MoIDPLH are subsidised for services and AP. Some fees exist for AP.	MoIDPLH scheme covers rehabilitation for all children under 3yo, children with disability up to 18yo, adults with disability, older people, war veterans and vulnerable population. UHCP has 95% population coverage (but rehabilitation not included). VHI schemes cover 6% of the population.	The MoIDPLH scheme includes treatment package with a pre-set number of treatments. E.g., eligible child receives approx. US $950.00 per year of services via maximum of 176 treatment sessions. AP is included in this scheme and vouchers can be used at approved service providers.
**8.** **JORDAN**	Fees exist for rehabilitation in government healthcare, the amount varies between facilities and services and based on the types of insurance or the residency status of recipients. Some fees exist for AP.	The tax-based public health scheme of MOH covers Jordanian citizens, in this way coverage is good. A high number of people have insurance, 68% of population is included.	The tax-based public health scheme finances the rehabilitation provided in hospitals, this includes the full scope of interventions provided by existing rehabilitation workers. The major health insurance schemes include the services of most rehabilitation professions, such as PT, OT, SLT and Psychology, the number of sessions is capped (e.g. 5–10 sessions), and the adjustment for severity of injury was described as inadequate with some people receiving insufficient dose. Funding for assistive products is included but limited supply (mostly mobility products).
**9.** **GUYANA**	No or minimal fees exist for rehabilitation in government healthcare. Some fees exist for AP.	The tax-based public health scheme of MOPH covers Guyana citizens, in this way coverage is good. The NIS covers approx. 3.5% of the population.	The tax-based public health scheme finances the rehabilitation provided in government hospitals, this includes the full scope of interventions provided by existing rehabilitation workers. Assistive products also included but limited in range (mostly mobility products) and quantity supplied, and fees are applied to AP.

### Rehabilitation information (Tables 6 and 7)

Information on rehabilitation from MOH administrative sources was limited. Specifically, workforce data was available in the MOH workforce accounts in Guyana, Solomon Islands and Sri Lanka; these countries also routinely collated information on availability of services, however the six other countries did not (Table 6). Expenditure data was incomplete or not available in all nine country’s national health accounts. Generally, health facilities in all countries collected service utilization data, however, in seven countries this was neither standardised or collated and reported at district or national levels, although this did occur in Guyana and Solomon Islands. No country routinely collected and reported service outcomes, population need or coverage of rehabilitation services (Table 7). Guyana and Solomon Islands, the two smallest countries by population, had the most comprehensive rehabilitation information available.

**Table 6:** Rehabilitation information.

COUNTRY	Rehabilitation information in MOH administrative sources: National Health Workforce Accounts (NHWA) and National Health Accounts (NHA)	Rehabilitation information in MOH administrative sources: Health services, including availability of rehabilitation across levels of healthcare and rehabilitation beds.	Facility level rehabilitation information available: Service utilization, quality and outcomes
**1. MYANMAR**	Not routinely available in MOHS workforce database or national health accounts. Workforce data was identified through the professional associations. Not available in NHA.	Not available in MOHS administrative database but existing rehabilitation services and beds were identifiable through professional associations and networks.	Information is collected in facilities, including cases, diagnosis and sessions, however not standardised, collated or reported at district or national level. Very limited information reflecting quality or outcomes is routinely collected.
**2.** **SRI LANKA**	Workforce data available, included number of workers for each profession in government healthcare. Some information available in NHA.	Yes. Routinely available for government healthcare through the Annual Health Statistics, this reports on number of rehabilitation workers (their profession and facility site) and number of rehabilitation beds. Newly introduced reporting on prosthetic and orthotic services.	Information is collected in facilities, including cases, diagnosis and sessions, however not standardised, collated or reported at district or national level. Very limited information reflecting quality or outcomes is routinely collected.
**3.** **NEPAL**	Not routinely available in MOHP workforce database, information available through the professional associations. Some information available in NHA but not complete set as expenditures integrated into curative care, not able to disaggregate.	Not available in MOHP administrative database but existing rehabilitation services and beds were identifiable through professional associations and government networks.	Information is collected in facilities, including cases, diagnosis and sessions, however this is not standardised, collated or reported at district or national level. Very limited information reflecting quality or outcomes is routinely collected.
**4.** **SOLOMON ISLANDS**	Workforce data available, including number of workers for each profession in government healthcare. Some information included in NHA but some expenditure integrated into curative care, not able to disaggregate.	Yes. Routinely available for government healthcare through annual health planning and reporting.	Information collected in facilities, including cases, diagnosis, sessions and AP provided, this information is sent monthly to national level and included in the internal MHMS reporting. Very limited information reflecting quality or outcomes is routinely collected.
**5.** **TANZANIA**	Not routinely available in MOH workforce database or the national health accounts. Workforce data was identified through the professional associations and government networks. Some information within NHA.	Not available in MOH administrative database but existing rehabilitation services and beds were identifiable through professional associations and government networks.	Information collected in facilities, including cases, diagnosis and sessions, however not standardised, collated or reported at district or national level. Very limited information reflecting quality or outcomes is routinely collected.
**6.** **ZAMBIA**	Not routinely available in MOH workforce database but some identified through the professional associations and government networks. Some data identified within NHA	Not available in MOH administrative database but existing rehabilitation services and beds were identifiable through professional associations and government networks.	Information collected in facilities, including cases, diagnosis and sessions, however not standardised, collated or reported at district or national level. Very limited information reflecting quality or outcomes is routinely collected.
**7.** **GEORGIA**	Not routinely available in MOH workforce database, incomplete workforce data was identified through professional networks. Some data identified within NHA.	Not available in MOH administrative database. Some (not full set) existing rehabilitation services and beds were identified through professional associations and government networks.	Information collected in facilities, including cases, diagnosis and sessions, however not standardised, collated or reported at district or national level. Very limited information reflecting quality or outcomes is routinely collected.
**8.** **JORDAN**	Not routinely available in MOH workforce database or the national health accounts. Incomplete workforce data was identified through networks, not able to use for the assessment.	Not available in MOH administrative database. Some (not full set) existing rehabilitation services and beds were identified through professional associations and government networks.	Information collected in facilities, including cases, diagnosis and sessions, however not standardised, collated or reported at district or national level. Very limited information reflecting quality or outcomes is routinely collected.
**9.** **GUYANA**	Workforce data available, including number of workers for each profession in government healthcare. Some data included in NHA but expenditure integrated into curative care, not able to disaggregate.	Yes. Routinely available for government healthcare through annual health planning and reporting.	Information collected in facilities, including cases, diagnosis, sessions and AP provided, this information is regularly sent to national level where included within internal MOPH reporting. Very limited information reflecting quality or outcomes is routinely collected.

**Table 7:** Rehabilitation information, cont’d.

	Rehabilitation needs can be inferred from national health and demographic data?	Information on population functioning available?	Information on population coverage of rehabilitation for specific population groups available?	Rehabilitation research
**1.** **MYANMAR**	yes	no	no	Limited. Small amount occurring in academic institutions, mostly during post-graduate training of rehabilitation medicine doctors.
**2.** **SRI LANKA**	yes	Information on population functioning was available based on WHO Model Disability Survey	no	Limited. Small amount occurring at academic institutions, mostly with international collaboration.
**3.** **NEPAL**	yes	no	no	Limited. Small amount occurring at academic institutions, mostly with international collaboration.
**4.** **SOLOMON ISLANDS**	yes	no	no	Not occurring.
**5.** **TANZANIA**	yes	no	no	Limited. Small amount occurring at academic institutions, mostly with international collaboration.
**6.** **ZAMBIA**	yes	no	No, but a 2015 disability survey estimated proportion of people who needed and didn’t receive rehab and AP, 63% and 73% respectively.	Not occurring.
**7.** **GEORGIA**	yes	no	no	Limited. Moderate amount occurring at academic institutions, mostly with international collaboration.
**8.** **JORDAN**	yes	no	no	Limited. Moderate amount occurring at academic institutions, mostly with international collaboration.
**9.** **GUYANA**	yes	no	no	Not occurring.

### Rehabilitation workforce (Tables 8–10)

Rehabilitation workforce information included national workers from government and private sectors as well as international workers and volunteers. All lower middle-income countries had five or less rehabilitation workers per 100,000 population, whereas upper middle income ranged from 12–22 per 100,000, reflecting a difference between country income levels (Table 8). Regarding total numbers of workforce, Jordan could not produce reliable data in their STARS report. The number of rehabilitation professions was lowest with four in Myanmar and Zambia and highest with seven in Nepal, international volunteers increased this in smaller countries such as Guyana and Solomon Islands. The median number of cadres for the upper middle-income countries was 6, and for lower middle-income countries it was 5. Physiotherapists, occupational therapists and prosthetists and orthotists were available in all countries and all countries had at least one training course for rehabilitation cadres with two of the upper middle-income countries having three or more post-graduate courses compared with none in four of the lower middle income (Table 9). Centralised workforce planning to allocate rehabilitation workers to government healthcare occurred in seven countries. Supervision practices of rehabilitation workers in government healthcare was characterised as basic with few systematic processes in place. Multiple common workforce challenges were reported across all countries (Table 10), regardless of income category, and these included; limited professional development opportunities (eight countries); lack of rehabilitation professions available (six countries); difficulty in attracting and retaining workers outside major urban areas (six countries); limited opportunity for career progression (five countries); low profile of rehabilitation professions; rehabilitation not being a popular career choice (three countries) and rehabilitation workers not feeling valued by other healthcare professionals (three countries).

**Table 8:** Rehabilitation workforce.

COUNTRY	Number of rehabilitation workers		Composition of rehabilitation workforce–total number of workforce and per 100,000 population (workforce density)							
	**Total** **&** **Total per 100,000 population**	**Rehab professions in country**	***Physiotherapists (PT)***	***Occupational Therapists (OT)***	***Speech and Language Therapists (SLT)***	***Prosthetists and Orthotists (P&O)***	***Psychologists (Psych)***	***Physical Rehabilitation Medicine Doctors*** ***(PRM)***	***Rehabilitation Nurses*** ***(RN)***	***Mid-level rehabilitation workers*** ***(MLRW)*** ****
**1. MYANMAR**	1764	4	1539	2	0	17	0	206	NA	Not an established cadre
	3.264 per 100k		2.848 per 100k	0.004 per 100k	0.000 per 100k	0.031 per 100k	0.000 per 100k	0.381 per 100k		
**2. SRI LANKA**	655	5	424	98	62	35	0	35*	0	Not an established cadre
	3.004 per 100k		1.945 per 100k	0.449 per 100k	0.284 per 100k	0.161 per 100k	0.000 per 100k	0.165 per 100k	0.000 per 100k	
**3. NEPAL**	1359	7	1200**	13	51	37	27	1	30	Not an established cadre
	4.750 per 100k		4.195 per 100k	0.045 per 100k	0.178 per 100k	0.129 per 100k	0.094 per 100k	0.003 per 100k	0.105 per 100k	
**4. SOLOMON ISLANDS**	35	5	8	4	1	2	0	0	0	20
	5.225 per 100k		1.194 per 100k	0.597 per 100k	0.149 per 100k	0.299 per 100k	0.000 per 100k	0.000 per 100k	0.000 per 100k	2.986 per 100k
**5. TANZANIA**	657	5	420	81	5	81	20	0	0	50
	1.133 per 100k		0.724 per 100k	0.140 per 100k	0.009 per 100k	0.140 per 100k	0.034 per 100k	0.000 per 100k	0.000 per 100k	0.086 per 100k
**6. ZAMBIA**	455	4	432	4	4	15	0	0	0	Not an established cadre
	2.547 per 100k		2.419 per 100k	0.022 per 100k	0.022 per 100k	0.084 per 100k	0.000 per 100k	0.000 per 100k	0.000 per 100k	
**7. GEORGIA**	840	6	500	25	*** Data not available	15	Data not available.	300	Data not available.	Not an established cadre
	22.578 per 100k		13.439 per 100k	0.672 per 100k		0.403 per 100k		8.064 per 100k		
**8. JORDAN**	Data not available.	6	Data not available.	Data not available.	Data not available.	Data not available.	Data not available.	Data not available.	Data not available.	Not an established cadre
**9. GUYANA**	98	5	22	5	5	2	0	0	0	64
	12.520 per 100k		2.811 per 100k	0.639 per 100k	0.639 per 100k	0.256 per 100k	0.000 per 100k	0.000 per 100k	0.000 per 100k	8.176 per 100k

* In Sri lanka is it agreed that Rheumatologists will undertake PRM role although the country has commenced training its first PRM.

** The Nepal PT professional association provided this number but also estimates that only 50% are actually working as PTs (many have immigrated or taken other work)

*** For comparability, SLT estimates are for people with SLT diploma, degree or masters in the country. Georgia’s data could not separate SLTs from logopeds (Special education trained professionals which undertake some SLT roles, approx. 1000 logopeds were reported).

**** Mid-level rehabilitation workers are those that have undertaken training in rehabilitation and deliver predominantly rehabilitation care but don’t have a qualification which is a diploma, degree or above. This typically includes community-based rehabilitation (CBR) workers or therapy assistants. CBR workers who were primarily focused on disability mainstreaming or disability-inclusive development, i.e. the education, livelihood, social and empowerment components of the WHO CBR matrix were not included.

Note—country population data was extracted from the World Bank country profiles.

**Table 9:** Rehabilitation workforce 2, cont’d.

	Rehabilitation profession training (degree or diploma) in national training institutions	Availability of postgraduate rehabilitation training in country	Features of the workforce distribution: Public/Private, Tertiary/Primary, Urban/Rural	Features of workforce planning and supervision
**1.** **MYANMAR**	3 (PT, PRM, P&O)	MSc & PhD in PRM MSc in PT	Majority in Public. Majority in Tertiary care. Majority in Urban area.	Centralised regular planning through central application and allotment to rehabilitation posts in government healthcare. Basic workplace supervisory practices in place.
**2.** **SRI LANKA**	4 (PT, OT, SLT, P&O)	No post-grad available	Majority in Public. Majority in Tertiary care. Majority in Urban area.	Centralised regular planning through central application and allotment to rehabilitation posts in government healthcare. Basic workplace supervisory practices in place.
**3.** **NEPAL**	3 (PT, OT, Psych)	MSc & PhD in Psych	Majority in Private (96%). With workers commonly located in private practice at primary and secondary care level Majority of government workers in Tertiary level. Majority urban and 76% located in Bagmati Province (greater Kathmandu area).	Recent centralised planning of workforce for government healthcare. Basic workplace supervisory practices in place.
**4.** **SOLOMON ISLANDS**	1 (MLRW—diploma level training)	No post-grad available	Majority in Public (100%). >90% in Tertiary care. > 90% in Urban area.	Centralised, regular planning for government healthcare. Centralised supervisory mechanism with the MHMS Rehabilitation and Physiotherapy Division supervising workers outside of the National Referral Hospital. Internship programme for MLRWs developed to build capacity for practice in isolated settings.
**5.** **TANZANIA**	3 (PT, OT, P&O)	No post-grad available	Small majority in Public (53%). Majority in government healthcare work in tertiary care. Majority in Urban area.	Centralised, regular planning for government healthcare. Basic workplace supervisory practices in place.
**6.** **ZAMBIA**	1 (PT)	No post-grad available	Majority in Public. Majority in Tertiary care. Majority in Urban area.	Centralised, regular planning for government healthcare. Basic workplace supervisory practices in place.
**7.** **GEORGIA**	6 (PRM, PT, OT, SLT, RN, Psych)	M Sc in OT MSc in PT MSc in Rehabilitation—multiple specialties MSc in SLT MSc in Psych	Majority in Private Information not available	Limited planning not centralised. No information about supervisory practices.
**8.** **JORDAN**	5 (PT, OT, SLT, P&O, PRM)	MSc in PT MSc in SLT MSc in Rehabilitation Science	Information not available.	Limited planning not centralised. No information about supervisory practices.
**9.** **GUYANA**	3 (PT, SLT, the MLRW is a certificate level programme)	No post-grad available	Majority in Public (95%) Majority in Tertiary care. Majority in Urban areas with moderate distribution across provinces	Centralised, regular planning for government healthcare. Basic workplace supervisory practices in place.

**Table 10:** Rehabilitation workforce, cont’d.

	Regulation and licensing of rehabilitation workforce	Commonly reported rehabilitation workforce challenges
**1.** **MYANMAR**	PRMs regulated and licenced under the Myanmar Medical Council. Plans underway for rehabilitation therapists to also be included.	◾ Limited professional development opportunities. ◾ Lacking key professions (For Myanmar—OT, SLT, Psych). ◾ Difficult to attract and retain workers outside major urban areas. ◾ Limited opportunity for career progression. ◾ Attrition from government healthcare, growing demand for workers in private sector. ◾ Government remuneration unattractive. ◾ Some institutional practices reduce therapist’s autonomy.
**2.** **SRI LANKA**	Only Doctors, PT and OT professions regulated through the Sri Lanka Medical Council.	◾ Limited professional development opportunities. ◾ Lacking key professions (For Sri Lanka—PRM, Psych). ◾ Difficult to attract and retain workers outside major urban areas. ◾ Limited opportunity for career progression. ◾ Attrition from government healthcare, growing demand for workers in private sector. ◾ Caseloads high in government healthcare resulting in client under-servicing. ◾ Limited infrastructure, equipment and consumables
**3.** **NEPAL**	Nepal Health Professionals Council regulates the Bachelor and Masters level therapists.	◾ Limited professional development opportunities. ◾ Difficult to attract and retain workers outside major urban areas. ◾ Limited opportunity for career progression. ◾ Few rehabilitation posts in government healthcare, highly privatised workforce, high number (esp. PTs) leave sector and or country. ◾ Rehabilitation has low profile and under-valued by other health workers. ◾ Limited infrastructure, equipment and consumables
**4.** **SOLOMON** **ISLANDS**	No licensing required	◾ Limited professional development opportunities. ◾ Lacking key professions (esp. OT, SLT, PRM). Reliance on international volunteers. ◾ Number of scholarships for therapy training is low, at risk of not enabling service growth or even sustaining the workforce. ◾ Often only one rehabilitation worker in facility which results in professional isolation and demotivation; ◾ MLRWs have limited access to transport and difficulty fulfilling planned community outreach role. ◾ Limited infrastructure, equipment and consumables
**5.** **TANZANIA**	Rehabilitation workers regulated under the Medical Council of Tanzania. By-laws regarding details of regulation yet to be developed.	◾ Limited professional development opportunities. ◾ Lacking key professions (For Tanzania SLT, Psych) ◾ Difficult to attract and retain workers outside major urban areas. ◾ Limited opportunity for career progression. ◾ Few rehabilitation posts in government healthcare ◾ Student intake low and at risk of not meeting growing demand from private sector. ◾ Rehabilitation has low profile and under-valued by other health workers.
**6.** **ZAMBIA**	Licensing required for rehabilitation workers through the National Health Professions Council. PTs need to complete 100 hours of continuing education to renew their licence.	◾ Limited professional development opportunities. ◾ Lacking key professions (For Zambia—OT, SLT, Psych, PRM). ◾ Difficult to attract and retain workers outside major urban areas. ◾ Limited opportunity for career progression. ◾ Attrition from government healthcare, growing demand for workers in private sector. ◾ Government remuneration unattractive. ◾ Student intake low and at risk of not meeting growing demand from private sector. ◾ Rehabilitation has low profile and under-valued by other health workers
**7.** **GEORGIA**	Only medical doctors are regulated and require a licence to practice. A working group has been established to identify professional standards and licencing mechanism for other rehabilitation workers.	◾ Rehabilitation workforce salaries are not standardised and negotiated with individual employers, risk of under-valuing. ◾ Concerns regarding variation in standards of rehabilitation workforce training and that some rehabilitation courses are not integrated into the National Centre for Educational Quality Enhancement (NCEQE).
**8.** **JORDAN**	All rehabilitation workforce must be licensed through the Directorate of Licensing Professions and Health Institution. Relicensing every 5 years now requires certain number of education hours. Additional license is required from the MOH to have a private practice.	◾ Limited professional development opportunities. ◾ Difficult to attract and retain workers outside major urban areas. ◾ Limited opportunity for career progression. ◾ Lacking planning and information regarding rehabilitation workforce. ◾ Student intake not aligned to market needs, sometimes over supply, many immigrate. ◾ Government remuneration unattractive, not in line with qualification. ◾ Some institutional practices reduce therapist autonomy.
**9.** **GUYANA**	All rehabilitation workers must attain their annual license from the Allied Health Professions Council, Guyana.	◾ Limited professional development opportunities. ◾ Lacking key professions (For Guyana—Psych, PRM). Reliance on international volunteers. ◾ Limited opportunity for career progression. ◾ Limited infrastructure, equipment or consumables

### Assistive products, and rehabilitation infrastructure and equipment (Table 11)

Eight MOHs intermittently undertook centralised procurement of AP. MOH procurement was driven by rehabilitation focal points in collaboration with medical equipment departments however this generally occurred in the absence of national guidance and specifications. Infrastructure and equipment typically varied across facilities (Table 11). No country had a comprehensive inventory or report on rehabilitation infrastructure and equipment, hence conclusions regarding availability across level of healthcare are cautious, however, typically, where high intensity, longer-stay rehabilitation facilities existed these had adequate levels of equipment and treatment spaces; similarly large tertiary hospitals had adequate equipment but often limited treatment spaces; secondary hospitals often had treatment spaces but inadequate equipment; government supported primary care rarely had rehabilitation infrastructure or equipment. The STARS reports from Guyana, Solomon Islands and Sri Lanka explicitly mentioned lack of infrastructure, equipment and componentry for prosthetics and orthotics.

**Table 11:** Assistive products, and rehabilitation infrastructure and equipment.

COUNTRY	Assistive technology procurement practices of the government agencies	Existing rehabilitation infrastructure and equipment in government healthcare, including national guidance or standards for facilities
**1.** **MYANMAR**	Regular centralised procurement through MOHS. A rehabilitation sub-committee sitting under the Central Regular Procurement Committee meets to determine the AP purchased for distribution to government healthcare. No national AP specifications to guide procurement were available.	Varies across facilities. Treatment spaces were satisfactory, as was equipment, evidence of recent investment. Equipment in tertiary hospitals better than secondary where services are still emerging. No government guidance or facility standards exist.
**2.** **SRI LANKA**	Regular centralised procurement occurs through MOHNIM. No national AP specifications to guide procurement were available.	Varies across facilities. Therapy treatment spaces were limited in tertiary hospitals compared with hospital bed numbers and therapist workload, sometimes space was reported as preventing further service delivery. Equipment also limited, prosthetic and orthotic equipment of particular concern in some facilities. National Guidelines for Services exist and provide guidance for infrastructure and equipment
**3.** **NEPAL**	Intermittent centralised procurement occurring through MOHP. Also occurs at local government units through funding accessed from the Ministry of Women, Children and Senior Citizens. The Nepal PAPL includes some specifications which have assisted procurement by local government.	Varies across facilities. Therapy treatment spaces were limited in tertiary hospitals. Equipment also limited. Very little available in secondary level. Lack some standardized assessment kits/templates, tend to use versions adapted for India. No government guidance or facility standards exist.
**4.** **SOLOMON ISLANDS**	Intermittent centralised procurement through National Medical Stores (NMS). The NMS Essential Medical Supplies List does not include AP section, only crutches and orthopaedic collars included. No national AP specifications to guide procurement were available.	Generally limited and in need of upgrade. Therapy treatment space in National Referral Hospital requiring investment, some electrotherapy modalities were lacking maintenance, prosthetics and orthotic department in poor condition. Provincial hospital therapy treatment spaces and equipment typically sparse and outdated. Lacking standardized assessment kits/templates and not adapted to country. No government guidance or facility standards exist.
**5.** **TANZANIA**	Intermittent centralised procurement occurring through Medical Stores Department (MSD). AT not included in the MSD catalogue. No national AP specifications to guide procurement were available.	Varies across facilities. Treatment spaces were satisfactory, as was equipment. Equipment in tertiary hospitals better than secondary, OT treatment spaces requiring attention. Lacking standardized assessment kits/templates and not adapted to country. The Basic Rehabilitation Standards for Health Facilities—Volume 6 provides guidance for infrastructure and equipment.
**6.** **ZAMBIA**	Intermittent centralised procurement occurring by MOH, mostly focused on mobility AP. No national AP specifications to guide procurement were available.	Varies across facilities. Treatment spaces were satisfactory, as was equipment. Some electrotherapy modalities were lacking maintenance. Equipment in tertiary hospitals better than secondary where services still emerging. No government guidance or facility standards exist.
**7.** **GEORGIA**	MoIDPLHSA does not undertake centralised procurement. Providers (often pharmacy/businesses) procure separately.	Varies across facilities. Treatment spaces were satisfactory, as was equipment, electrotherapeutic equipment commonly available. Therapeutic swimming pools present in a few facilities. No government guidance or facility standards exist.
**8.** **JORDAN**	All three public sector health service delivery agencies (MOH, RMS and Universities) procure different AP for their constituents. No national AP specifications to guide procurement were available.	Varies across facilities. Treatment spaces were satisfactory, as was equipment although OT services did not have adequate splinting supplies to cover need. No government guidance or facility standards exist.
**9.** **GUYANA**	Intermittent centralised procurement by MOPH, mostly focused on mobility AP. No national AP specifications to guide procurement were available.	Varies across facilities but characterised as limited and in need of upgrade. Therapy treatment space often small and equipment sparse and outdated. Prosthetics and orthotic equipment in need of investment. Lacking standardized assessment kits/templates and not adapted to country. No government guidance or facility standards exist.

### Rehabilitation services (Tables 12–14)

High intensity, longer stay rehabilitation, required for people with complex needs was very limited across all countries with rehabilitation beds per capita ranging from 1 per 43,000 Sri Lanka to 1 bed per 2.4 million in Tanzania; country income category did not align with rehabilitation bed per capita. Rehabilitation services were mostly available in tertiary level government hospitals, with 100% availability in tertiary hospitals in Guyana, Nepal, Solomon Islands and Zambia, a percentage value was not available for Jordan and Georgia. Physiotherapy services were most frequently available across all countries. Availability of rehabilitation reduced considerably in secondary care, with upper middle-income countries reporting a slightly higher mean rehabilitation availability at this level than that in lower middle-income countries. Availability of rehabilitation in government supported primary care was almost non-existent with only Myanmar and Solomon Islands reporting limited availability (Table 12). All countries had significantly less rehabilitation services outside major urban areas. Early identification and referral of children in need of rehabilitation was mostly characterised as emerging or in need of establishment in all countries. Integration of rehabilitation into mental health care was established but limited to psychology and occupational therapy services in specialist mental health facilities. Integration of rehabilitation into eye care and hearing care was assessed by availability of low vision services and audiology services that provided hearing aids, respectively (Table 13). Rehabilitation for eye care was very limited in four countries (only in 1–2 sites), whereas Sri Lanka reported access across multiple government sites and Georgia reported many private providers of eye care; rehabilitation for hearing care was very limited in five countries, with the private sector providing hearing aids in seven countries. All countries had non-government organizations (NGOs) delivering some rehabilitation for people with disabilities in community settings, these services also commonly provided social supports, education and livelihood focused programmes; in all countries, the extent to which these services were available across the entire country was estimated to be low. The provision of AP was dominated by mobility with some vision and hearing products available, AP targeting people’s communication and cognition was particularly low with four countries not providing any such AP (Table 14).

**Table 12:** Rehabilitation services 1.

COUNTRY	High intensity, longer-stay, specialised rehabilitation available for people with complex needs (number of physical rehab beds per capita)	Rehabilitation available in tertiary healthcare (percentage of tertiary hospitals with rehabilitation services)	Rehabilitation available in secondary healthcare (percentage of secondary hospitals with rehabilitation services)	Rehabilitation available in government primary care (percentage of primary care with rehabilitation services)
**1. MYANMAR**	1 government national rehabilitation hospital in Yangon. Total of 233 beds, 1 bed to 219,000 people	79% of tertiary hospitals included either a physical medicine and rehabilitation (PMR) department or physiotherapy unit	76% of secondary level hospitals have either a PMR department or a physiotherapy unit.	16% of the Township hospitals (considered highest level of PHC services) had physiotherapy unit. No rehabilitation in PHC facilities below this level.
**2.** **SRI LANKA**	1 government national rehabilitation hospital (Ragama), and 5 other government units/centres with rehabilitation bed capacity. Total of 490 beds, 1 bed to 43,000 people	95% have physiotherapy, 57% have occupational therapy, 47% speech language therapy, 35% prosthetic and orthotic services	38% have physiotherapy, 6% occupational therapy 10% speech language therapy.	0% of government PHC includes therapy services
**3.** **NEPAL**	No dedicated government rehabilitation facility, multiple centres, units ran by NGOs, a few with small government financial support. Total of 300 beds 1 bed per 87,000 people	100% have physiotherapy 4% speech, language and audiology services. 0% with occupational therapy, prosthetics and orthotics.	7% have physiotherapy.	0% of government PHC includes therapy services. (Private service providers in urban areas commonly deliver care at this level).
**4.** **SOLOMON ISLANDS**	No dedicated government rehabilitation facilities, beds at National Referral Hospital used for rehabilitation patients for an extended stay.	100% of tertiary care with rehabilitation as only one National Referral Hospital in country, this has PT, OT, SLT and P&O services.	33% of secondary care have physiotherapy services. Out of 12 Provincial /General Level Hospitals, 4 have physiotherapy. A few also have CBR worker services.	20 CBR workers in the country offer some rehab in PHC as well as in community. Some CBR workers based in larger PHC Health Centres
**5.** **TANZANIA**	No dedicated government rehabilitation hospital or centre. Beds in tertiary hospitals used for rehabilitation patients for an extended stay. One 24 bed specialised spinal cord injury ward in government hospital. 1 bed per 2.4 million people	Rehabilitation is generally available in tertiary hospitals but data was not available.	Rehabilitation is characterised as emerging in secondary hospitals but data not available.	Rehabilitation is not integrated into government delivered PHC, there are isolated examples of PTs from secondary or tertiary level undertaking outreach into larger PHC facilities. Private service providers in urban areas commonly deliver care in clinics or homes.
**6.** **ZAMBIA**	No dedicated government rehabilitation hospital, centre or ward. Beds in tertiary hospitals provided to rehabilitation patients for an extended stay. Government estimates approx. 50 beds in government hospitals are utilised for rehabilitation for complex clients at any one time, if so this is 1 bed per 357,000 people.	Rehabilitation is available in 100% of government tertiary hospitals.	Rehabilitation is available in 100% of government secondary hospitals.	0% of government PHC includes therapy services
**7.** **GEORGIA**	Specialised rehabilitation available within government centres including the Lung Diseases Rehabilitation Centre and National Hero of Maro Centre, however most services in private healthcare. Bed number unavailable.	Rehabilitation is common in tertiary level care but information and data was not available.	Information and data was not available.	Not included in government supported PHC.
**8.** **JORDAN**	One dedicated centre, the Royal Jordanian Rehabilitation Centre, which is part of the Royal Medical Services, this facility has 41 beds. Beds in other tertiary hospitals are provided to rehabilitation patients for an extended stay. 1 bed for 250,000 people	Rehabilitation available in: 16 out of 32 Ministry of Health tertiary and secondary hospitals, 8 out of 17 Royal Medical Hospitals, 3 out of 3 University Hospitals. These include both tertiary and secondary care. 52% of the total tertiary and secondary hospitals have rehabilitation.	Tertiary and secondary care reported together. See cell to left.	0% of the government PHC includes therapy services
**9.** **GUYANA**	No dedicated government rehabilitation hospital, centre or ward. Beds in tertiary hospitals used for rehabilitation patients for an extended stay.	100% of tertiary care hospitals have rehabilitation. The Georgetown Public Hospital has PT, OT, SLT and audiology services.	100% of secondary care hospitals have rehabilitation.	Rehabilitation is not integrated into government delivered PHC. Private service providers in urban areas commonly deliver care at this level.

**Table 13:** Rehabilitation services, cont’d.

	Rehabilitation delivered in community settings	Rehabilitation for children—including identification, community-based early childhood intervention, services within tertiary and secondary hospitals and services delivered within education settings.	Rehabilitation available within mental health, vision and hearing care
**1.** **MYANMAR**	Limited provision by government healthcare, occasionally home visits occur. Local government utilises community-based rehabilitation for people with disabilities, addresses rehabilitation among other areas such as accessibility of the physical environment. Development partners support some NGOs with focus on people with disabilities.	Early identification and referral practices are under-developed across healthcare. Community based early childhood intervention is emerging, a new initiative led by Ministry of Social Welfare, Relief and Resettlement is underway to build early childhood intervention programmes.Four children’s hospitals exist, all with rehabilitation services. There are no government supported programmes that provide rehabilitation (e.g. therapy services) in mainstream or special education settings.	Limited rehabilitation in specialised mental health care, no OTs present, limited community level mental health care. Limited rehabilitation in eye care, one low vision clinic exists in the national eye hospital (through development partner support). Limited rehabilitation in hearing care, one audiology service provides hearing aids in the national ear hospital. A few private audiology clinics exist in the major urban areas.
**2.** **SRI LANKA**	Provision by government healthcare occurs in some provinces through therapists conducting outreach visits, sometimes with a multi-discipline mobile team or therapy home visit. Development partners support various projects, mostly focused on people with disabilities. MOSSPI support a CBID programme which may include some small components of rehabilitation.	Early identification practices are established across healthcare however referral and service delays are common. Community based early childhood intervention is mostly available through NGOs and private (for profit) services. The specialist children’s hospital in Colombo and some tertiary hospitals provide a comprehensive multi-discipline assessment and treatment programme. No government rehabilitation (e.g. therapy services) in mainstream or special education settings, some NGOS facilitate this.	Some rehabilitation is integrated into specialised mental health care, OTs regularly work in these services. Some rehabilitation is integrated into eye care, low vision clinics and provision of AP in multiple eye health services. Some rehabilitation is integrated into government supported hearing care but limited. A few private audiology clinics exist in the major urban areas.
**3.** **NEPAL**	No provision by government healthcare. Development partners support NGOs that mostly focus on people with disabilities. (Private service providers commonly deliver care in people’s homes)	Early identification and referral practices are under-developed across healthcare. Limited community based early childhood intervention exists, no government supported programmes, some NGO and private (for profit) services Two children’s hospitals exist and provide rehabilitation, other tertiary or secondary hospitals do not have specialised programmes for children although some rehabilitation provided. No government supported rehabilitation (e.g. therapy services) in mainstream or special education settings.	Limited rehabilitation in specialised mental health care, a few OTs present, limited community level mental health care. Limited rehabilitation in eye care, some low vision clinics in tertiary eyecare services. Limited rehabilitation in hearing care. A few private audiology clinics exist in the major urban areas.
**4.** **SOLOMON ISLANDS**	Established government supported community-based rehabilitation (CBR) programme with mid-level rehabilitation workers. 20 CBR Field Officers and CBR Aides are employed across all but one of the 9 provinces.	Early identification is under-developed across healthcare and referral and service delays are common. Community based early childhood intervention can be provided by MHMS CBR workers, also through NGOs. The tertiary hospitals provide children’s services however only multi-discipline assessment ands services are available at the national referral hospital. No government supported rehabilitation (e.g. therapy services) in mainstream or special education settings, some NGOs facilitate this.	Limited rehabilitation in mental health care, no specialised programmes/personnel (e.g. OTs) in place, CBR workers offer limited services in community settings. Limited rehabilitation in eye care, no low vision clinics nor provision of AP. No rehabilitation in hearing care, no audiology services, previous training of personnel but hearing aids provision not occurring.
**5.** **TANZANIA**	No provision by government healthcare, occasionally home visits occur. Development partners and government support various NGOs and projects that focus on people with disabilities. Private service providers may deliver care in people’s homes.	Early identification and referral practices are under-developed across healthcare. Early childhood intervention is mostly provided by NGOs and often this is centre-based, services delivered in homes are less available. Tertiary hospitals integrate rehabilitation into paediatric care but lacking some specialists such as SLTs. No government supported rehabilitation (e.g. therapy services) in mainstream or special education settings.	Some rehabilitation is integrated into specialised mental health care, OTs regularly work in these services but psychologists not available. Limited rehabilitation is integrated into eye care, a low vision services exists in one tertiary service (CCBRT). Limited rehabilitation is integrated into hearing care. Small number of private audiology clinics exist in the major urban areas.
**6.** **ZAMBIA**	No provision by government healthcare, occasionally home visits occur. Development partners support NGOs that focus on people with disabilities. Private service providers commonly deliver care in people’s homes.	Early identification and referral practices are under-developed across healthcare. Early childhood intervention is mostly provided by NGOs and often based from centres or through NGO supported CBR/CBID programmes. No government supported rehabilitation (e.g. therapy services) in mainstream or special education settings.	Limited rehabilitation is integrated into specialised mental health care, OTs in these services but psychologists not available. Limited rehabilitation is integrated into eye care, a low vision services exists in one tertiary hospital (UTH). Limited rehabilitation is integrated into hearing care. Small number of private audiology clinics exist in the major urban areas.
**7.** **GEORGIA**	Government financed programmes for children with severe disabilities support home-based care. Some rehabilitative care is delivered by nurses through a home-care service programme.	Early identification practices are established across healthcare. An extensive network of early childhood intervention service providers offer services in homes, early childhood programmes and specialised centres. Day-care centres for vulnerable children and school aged children with severe disabilities exist.	Assessment not inclusive of mental health. MoIDPLHSA programme funds some hearing aids, cochlear implants and vision AP. Assessment did not include rehabilitation within vision and hearing care.
**8.** **JORDAN**	Limited provision by government healthcare, occasionally home visits occur. Development partners support local NGOs, mostly focus on people with disabilities and include other disability support services.	Early identification and referral practices are established across maternal and child healthcare, and some regions have specialist centres for further assessment and diagnosis of children (referred to as early detection centres). Early childhood intervention services are provided by a mix of MOH, RMS and University hospitals, NGO and Ministry of Social development services. Government supports care to school aged children and some programmes transitioning Ministry of education. Two children’s hospitals exist with modest rehabilitation department offering some specialised multi-disciplinary care.	Rehabilitation integrated into mental health care albeit with further development required. National Mental Health Centre has OT, counselling and psychology services, an estimated 20 psychiatrists and 41 psychologists employed in MOH mental health services. Information not available for rehabilitation within vision and hearing care.
**9.** **GUYANA**	No provision by government healthcare, occasionally home visits occur. Development partners support NGOs that focus on people with disabilities. Private service providers commonly deliver care in people’s homes.	Early identification and referral practices are under-developed across healthcare. Community based early childhood intervention provided by the CBR workers and a few NGOs. Multi-discipline assessment and services available at Georgetown Public Hospital and one specialised centre (Ptolemy Reid). Government not supporting rehabilitation (e.g. therapy services) in mainstream or special education settings, some NGOs facilitate this.	Limited rehabilitation in specialised mental health care, no OT services, limited community level mental health care. Limited rehabilitation in eye care, no low vision clinic/services exist. Some rehabilitation in hearing care, audiology services exist (with 26 trained audiology technicians and 3 ear mould technicians) in hospitals and hearing aids are provided, funded by donor.

**Table 14:** Rehabilitation services, cont’d.

	Assistive products available within government healthcare, described by common categories of Assistive Products	Reported features that indicate delivery of quality rehabilitation care*	Reported areas of concern regarding quality of rehabilitation delivered**
**1.** **MYANMAR**	**Mobility**: Limited. Some orthopaedic collars, splints, braces, walking aids, white canes, quadripods, walking frames, crutches, wheelchairs, prosthetics and orthotics. **Vision**: Very limited. Small number of magnifiers & telescopes provided in National Eye Hospital through development partner support. **Hearing:** Very limited. National Ear Hospital provides approx. 50 hearing aids per year. **Communication and cognition:** None provided. **Environment**: None provided	Small number national clinical practice guidelines No documented referral procedures No routine use of outcome measures	Regular utilization of non-evidence-based interventions Low rehabilitation dose and intensity, limited follow-up Limited scope of interventions available Delayed care, especially in and from community settings Goal setting and multidisciplinary care mostly limited to specialised rehabilitation settings Limited use of person centred, flexible, tailored care
**2.** **SRI LANKA**	**Mobility:** Limited. Some orthopaedic collars, splints, braces, walking frames, crutches, seating support, wheelchairs, prosthetics and orthotics. **Vision:** Some. Magnifiers & telescopes provided in multiple eyecare services. **Hearing:** Limited. Some provided in hearing care programmes. **Communication and cognition:** Very limited. Small number provided in specialised rehabilitation services. **Environment:** Very limited. Small number provided in specialised rehabilitation services. In Sri Lanka, there is potential for individuals with disabilities to utilize government allowance and purchase their AP in local businesses.	No national clinical practice guidelines No documented referral procedures No routine use of outcome measures	Regular utilization of non-evidence-based interventions Low rehabilitation dose and intensity, limited follow-up Limited scope of interventions available Delayed care, especially in and from community settings Goal setting and multidisciplinary care mostly limited to specialised rehabilitation settings Limited use of person centred, flexible, tailored care
**3.** **NEPAL**	**Mobility:** Very limited. New national health insurance fund includes a few post-surgical orthopaedic products. Some prosthetics and orthotics provided outside of MOH services. **Vision**: Very limited. Small numbers of magnifiers & telescopes provided in eye hospitals with development partner support. **Hearing:** None provided. **Communication and cognition**: None provided. **Environment:** None provided. In Nepal, there is potential for local governments to utilize disability budgets to procure AP and work with local health providers.	No national clinical practice guidelines No documented referral procedures No routine use of outcome measures	Regular utilization of non-evidence-based interventions Low rehabilitation dose and intensity, limited follow-up Limited scope of interventions available Delayed care, especially in and from community settings Goal setting and multidisciplinary care mostly limited to specialised rehabilitation settings Limited use of person centred, flexible, tailored care
**4.** **SOLOMON** **ISLANDS**	**Mobility**: Limited. Some splints, braces, walking frames, crutches, seating support, wheelchairs, prosthetics and orthotics. **Vision:** None provided. **Hearing**: None provided. **Communication and cognition:** None provided. **Environment:** Very limited. Small number provided in rehabilitation to enable return home after significant injury/disease. In Solomon Islands, many AP and especially mobility products, are supplied by development partners for delivery in government healthcare.	No national clinical practice guidelines No documented referral procedures No routine use of outcome measures	Regular utilization of non-evidence-based interventions Low rehabilitation dose and intensity, limited follow-up Limited scope of interventions available Delayed care, especially in and from community settings Goal setting and multidisciplinary care mostly limited to specialised rehabilitation settings Limited use of person centred, flexible, tailored care
**5.** **TANZANIA**	**Mobility:** Limited. Some splints, braces, walking frames, crutches, wheelchairs, prosthetics and orthotics provided, and approx. 10 mobility AP included in the NHIF. **Vision:** Very limited, provided in eye care only when development partners have supported. **Hearing:** Very limited, can access through NHIF. **Communication and cognition**: Very limited, provided in specialised rehabilitation when development partners have supported. **Environment:** None provided.	No national clinical practice guidelines No documented referral procedures No routine use of outcome measures	Regular utilization of non-evidence-based interventions Low rehabilitation dose and intensity, limited follow-up Limited scope of interventions available Delayed care, especially in and from community settings Goal setting and multidisciplinary care mostly limited to specialised rehabilitation settings Limited use of person centred, flexible, tailored care
**6.** **ZAMBIA**	**Mobility:** Very limited. Some splints, braces, walking aids, walking frames, crutches, wheelchairs, prosthetics and orthotics. **Vision:** None provided. **Hearing:** None provided. **Communication and cognition:** None provided. **Environment:** None provided In Zambia, almost all AP must be purchased privately or is provided through development partner support.	No national clinical practice guidelines No documented referral procedures No routine use of outcome measures	Regular utilization of non-evidence-based interventions Low rehabilitation dose and intensity, limited follow-up Limited scope of interventions available Delayed care, especially in and from community settings Goal setting and multidisciplinary care mostly limited to specialised rehabilitation settings Limited use of person centred, flexible, tailored care
**7. GEORGIA**	**Mobility:** Available. A wide range is funded, including electric wheelchairs. **Vision:** Available. **Hearing**: Available. **Communication and cognition:** Available. **Environment:** Available. In Georgia, the MoIDPLHSA funds 11 types of AP for people who are registered with government as person with a disability. Individuals are provided with a voucher and then go to approved providers to attain their AP.	Small number national clinical practice guidelines No documented referral procedures No routine use of outcome measures	Regular utilization of non-evidence-based interventions Low rehabilitation dose and intensity, limited follow-up Limited scope of interventions available Delayed care, especially in and from community settings Goal setting and multidisciplinary care mostly limited to specialised rehabilitation settings Limited use of person centred, flexible, tailored care
**8.** **JORDAN**	**Mobility:** Limited, although varies. Some splints, braces, walking frames, crutches, seating support, wheelchairs, upper and lower limb prosthetics and orthotics are available and on occasion high quality products are provided. Information lacking regarding vision, hearing, communication, cognition and environment products was not available. Some hearing aids are provided. In Jordan, multiple government agencies provide AP, detailed information was not available.	No national clinical practice guidelines No documented referral procedures No routine use of outcome measures	Regular utilization of non-evidence-based interventions Low rehabilitation dose and intensity, limited follow-up Limited scope of interventions available Delayed care, especially in and from community settings Goal setting and multidisciplinary care mostly limited to specialised rehabilitation settings Limited use of person centred, flexible, tailored care
**9.** **GUYANA**	**Mobility:** Limited. Some splints, braces, walking frames, crutches, seating support, wheelchairs, prosthetics and orthotics. **Vision:** Very limited. Low vision products through development partner support, spectacles are covered every 2 years by government. **Hearing:** Limited. Audiology services exist and through development partner support hearing aids are provided. **Communication and cognition:** None provided. **Environment:** None provided. In Guyana, much of the AP is supplied by development partners for delivery in government healthcare.	No national clinical practice guidelines No documented referral procedures No routine use of outcome measures	Regular utilization of non-evidence-based interventions Low rehabilitation dose and intensity, limited follow-up Limited scope of interventions available Delayed care, especially in and from community settings Goal setting and multidisciplinary care mostly limited to specialised rehabilitation settings Limited use of person centred, flexible, tailored care

*These items were selected because they can be observed or measured through being empirical in nature. They do not include workforce features that may indicate quality (e.g. licencing, training) as this is reported under workforce.

*For Clinical Practice Guidelines*: These are defined as nationally agreed documents based on evidence that guide decisions regarding care, they may be guidelines for comprehensive management of a health condition that integrate rehabilitation or specifically for rehabilitation. Measure used here was the availability of a national version.

*For Referral Procedures*: These are defined as common or standardised guidance for referral processes between services, these could be integrated into national healthcare referral guidance/procedures or developed specifically for rehabilitation. Measure used here was that they existed, were documented (somewhere) and promoted for use.

*For Outcomes Measures*: These are defined as common or standardised approaches to measure service outcomes, this could be routine, regular use of the same outcome measures for similar types of rehabilitation services or similar types of health conditions. A positive response requires a widely agreed and or documented set of outcomes measures that are widely utilised.

**These items were described in either the narrative/descriptive component of the STARS report or through the rating utilized in the rehabilitation maturity model, with grading that was either ’needs establishing’ or ’needs major strengthening’ considered a concern.

Quality of rehabilitation was difficult to assess at a national level as few empirical or measurable items were collated across facilities, also variation between facilities in countries can be high. The minimal comparable information reported in STARS included; existence of national clinical practice guidelines; wide-spread utilisation of documented outcomes measures; and nationally documented referral pathways. Of these, no country had all three and seven countries had none. Country reports also included other concerns regarding quality of services, including; regular utilization of non-evidence-based rehabilitation interventions; limited scope of rehabilitation with little specialisation of rehabilitation workers; low rehabilitation dose and intensity with limited follow-up, particularly when client discharged from hospital back to community; delays in receiving rehabilitation, particularly when client coming from community settings and especially for children with disabilities and developmental delays; limited availability of multidisciplinary, coordinated rehabilitation, which was mostly limited to specialised rehabilitation settings; and, limited use of person centred care in rehabilitation services.

## Discussion

In line with available evidence [17–19], our variable-oriented comparative study confirms and expand the evidence pointing out to a limited integration of rehabilitation into health systems in middle income countries. Some but not large distinctions between upper and lower middle-income country groups were observed, including for instance much lower numbers of rehabilitation workers per 100,000 population and slightly lower rehabilitation availability in secondary care in the latter. Governance and financing are mostly established but weak while integration of rehabilitation data in health information systems (HIS) is emerging and very weak. Rehabilitation workforce per capita is low, with considerable differences between countries and frequent reports of workforce challenges. Most countries experience extremely low availability for high-intensity rehabilitation, modest availability in tertiary hospitals, and low availability at the secondary, primary care and community level. Services outside of major urban areas are sparse. Provision of AP is limited with low levels of financing and high OOPE in all countries, and inadequate rehabilitation infrastructure is frequently experienced. As many of these findings and the challenges experienced in countries are relevant for other LMICs, these are now discussed in light of relevant literature and considering policy and practice implications which may be used to inform governments, and regional and global agencies about ways forward to strengthen rehabilitation in health systems.

### Governance

Governance weaknesses are not uncommon in health systems [20]. However, the limited national leadership, planning, coordination mechanisms, administrative capacity and accountability for rehabilitation observed across all countries is concerning. Contributing factors to this situation in LMICs include the historical focus on preventive and curative care [21], misperceptions of rehabilitation as a ‘disability service’ only [1], lack of recognition of what is rehabilitation because it is frequently called something else (e.g. physiotherapy) [22], and the often under-valued and fragmented workforce lacking critical mass and cohesion [23, 24]. Even in countries where planning for rehabilitation existed, this did not necessarily mean these plans were well-conceived and/or implemented. Appreciating that effective leadership and governance for rehabilitation is crucial in driving change, development partners and governments must focus on equipping relevant rehabilitation stakeholders within countries for this role. A practical way forward includes expanding rehabilitation leadership and administrative capacity at national and sub-national levels and developing multi-stakeholder national coordination mechanisms and sectoral networks, both good practice approaches [25]. Additionally, an important step in countries has been to undertake national rehabilitation strategic planning based on accurate insights into the country situation. While rehabilitation strategic planning may seem an unnecessarily ‘vertical planning approach’ for a health service that should be highly integrated into a wide range of healthcare, the process of bringing stakeholders together, discussing priorities, creating a shared vision, identifying objectives and then sequencing actions has been crucial for the sector’s efforts to advocate, coordinate, collaborate and importantly integrate rehabilitation across other relevant areas of health policy and planning [26].

The success of many efforts to strengthen rehabilitation will be underpinned by the ability to generate adequate rehabilitation data. For example, governance cannot be effective without information that informs decision making and enables accountability; and integrating rehabilitation into health benefit packages requires data to measure needs, utilization and outcomes. As reported previously [27], the current study shows that rehabilitation is not well integrated into health information systems, that generation of information is generally very limited and standardisation of rehabilitation data within and across countries is particularly low. Arguably some of the more politically influential data for decision making is where the greatest gaps exist, for example the financial expenditure for rehabilitation, utilisation rates, service outcomes and effective population coverage.

### Health information systems

There is an urgent need to address these challenges, especially considering that health decision making is occurring in an environment of increasingly competing needs for constrained resources. Fortunately, the integration of rehabilitation into HISs can be well guided by the opportunities and practices that already exist for other areas of healthcare. Ways forward for rehabilitation include using the WHO International Classification of Functioning, Disability and Health as a common language framework, as well as inclusion of rehabilitation information in MOH administrative data, such as the national health accounts and national workforce accounts; inclusion of rehabilitation data in routine health management information systems; and inclusion of rehabilitation into population surveys for monitoring effective service coverage. The WHO is already working on initiatives and products that address these areas, such as WHO’s Guidance on the Analysis and Use of Routine Health Information System, Rehabilitation Module [20] which provides a standardised data set for clinical facility use. Another example is the Rehabilitation Needs Estimator which harnesses GBD data for understanding rehabilitation needs, rather than promoting costly dedicated surveys [3]. In the health sector and other sectors, there is a frequently quoted saying—‘what gets measured gets done’, the findings from this study serve as a wakeup call to governments to begin measuring rehabilitation as a critical step in the process of strengthening rehabilitation in health systems.

### Rehabilitation services

Making rehabilitation services available in LMICs will also require further integration of rehabilitation into health financing schemes and health benefit packages. However, to achieve this several underlying challenges need attention. One challenge already mentioned is data, as countries increasingly move towards priority setting of health benefit packages that is more systematic, evidence-based and transparent [28], the need for better rehabilitation data increases. Another challenge, evident in this study, was that decisions to expand financing for any health service are generally informed by the existing availability of that service, which for example can be a barrier to the financing for rehabilitation in primary care, where it is particularly limited in LMICs [9]. Of the five countries with essential health benefit packages, rehabilitation was not included in three because it was not a service already available at this level. While this approach may seem understandable, it creates a chicken and egg situation that will require targeted efforts to address. This is being tackled in Myanmar, for example, where the rehabilitation workforce and services in primary care in one geographic location are being expanded with the explicit intention to demonstrate how in future, rehabilitation in primary care can become part of the country’s essential heath benefit package [29].

### Workforce

Responsive health systems rely on a well-trained workforce available at sufficient scale. The findings from this study in upper and low middle income countries align with studies of workforce density that have illustrated large differences across HICs and LMICs [28]. A large number of challenges existed for the rehabilitation workforce in countries in the present study, some of which were experienced more intensely by this workforce than others, for example, feeling misunderstood and under-valued by other health workers [24]. Typically, strengthening workforce means expanding trained workers, establishing new rehabilitation professions (e.g., speech and language therapists) and ensuring training is aligned with international standards and population needs. The findings suggest that training efforts should also include expanding rehabilitation in medical and nursing training, as the lack of this likely contributes to low levels of understanding and valuing of rehabilitation in health systems. Improving regulation is also an important lever for increasing recognition of the workforce and improving and monitoring quality. To assist countries, WHO has developed the Rehabilitation Competency Framework [30] and is currently piloting the WHO Guidance of Rehabilitation and Workforce and Evaluation [31], a tool to support a deep dive analysis into rehabilitation workforce within countries to inform more detailed workforce planning.

### Rehabilitation and primary care

While significant gaps in rehabilitation services exist across all levels of healthcare in many LMICs, expanding availability in primary care has the most potential to transform access for the population. Primary care is provided close to where people live and work, it reduces significant barriers to access such as transportation costs and time to service [28, 31, 32]. The renewed, global focus on PHC [33–35], the appreciation of its role in achieving Universal Health Coverage [36], and the investment occurring [37], make it a clear target of rehabilitation strengthening efforts. This is not without significant challenges because in LMICs there is limited availability of workforce and funding for the services. To address this, task-sharing approaches used in other areas of health care may be usefully employed [38, 39]. Currently, WHO is investing in the development of a resource that supports delivery of a limited set of rehabilitation interventions by existing primary care workers using a task-sharing approach, its purpose is to build capacity of existing primary care workers to identify rehabilitation needs, provide basic rehabilitation care and referral as needed. In an environment of constrained resources, decisions to expand services in one level may come at the cost of another, difficult decisions are required but should consider the potential primary care has at transforming access and subsequently achieve better population health and functioning outcomes [9].

### Assistive products

Assistive products primarily target human functioning [40]. Providing APs is one type of rehabilitation intervention in services characterised as physical, vision, hearing, cognitive or mental health rehabilitation services [41]. The limited inclusion of APs in financing mechanisms and resultant poor availability requires urgent attention. Substantial OOPE was reported by countries, and this hindered access and can trigger catastrophic health expenditure putting a strain on persons in need and their families. WHO, along with other development partners is working to address key challenges, including synthesising evidence through development of a Global Report on Assistive Technology [42], promoting streamlined and standards-based procurement [43] and supporting global partnerships to address assistive technology such as the AT scale Global Partnership.

The study had several limitations. First, the nine countries were all middle-income countries and had a MOH that had sought WHO support for strengthening rehabilitation in their health systems. The findings therefore cannot be generalised to all middle or low-income countries. Additionally, the relatively small number of upper middle-income countries in the study makes the findings about any distinction between upper and lower middle incomes countries tentative. Limited completeness of information by some countries also meant comparisons were descriptive rather than numerical in some instances. Nevertheless, this study is one of the first using a standardised assessment of rehabilitation using health policy and systems research and as such shines a light on gaps and challenges faced by middle-income countries.

### Conclusion

This variable oriented HSPR comparative study adds to the literature by providing for the first-time comprehensive information about rehabilitation across nine middle income countries, shedding light on the limited integration of rehabilitation in health systems and common areas of difficulty and challenge. It found that all countries had a basis on which rehabilitation strengthening efforts could be built, and that systematic analysis, as it is done in STARS, provides a good starting point from which to identify actions that need to be taken at national levels, as well as inform development of regional or global programmes and projects and policy and practice guidance. Additionally, our study can serve as a model for future studies, and by June 2022 a total of 26 countries have commenced STARS highlighting the potential for a larger cross-country comparative study including low-income countries.
